# Response to Durability of zuranolone in postpartum depression *versus* major depressive disorder

**DOI:** 10.1111/pcn.70076

**Published:** 2026-05-20

**Authors:** Masaki Kato, Kazuyuki Nakagome, Takamichi Baba, Takuhiro Sonoyama, Hiroki Fukuju, Ryosuke Shimizu, Juan Carlos Gomez, Tomoko Motomiya, Takeshi Inoue

**Affiliations:** ^1^ Department of Neuropsychiatry Kansai Medical University Osaka Japan; ^2^ Department of Psychiatry National Center of Neurology and Psychiatry Tokyo Japan; ^3^ Drug Development and Regulatory Science Division, Shionogi & Co., Ltd. Osaka Japan; ^4^ Shionogi B.V. London UK; ^5^ Department of Psychiatry Tokyo Medical University Tokyo Japan; ^6^ Sapporo Hanazono Hospital Hokkaido Japan

## Abstract

This article relates to Response to Durability of zuranolone in postpartum depression *versus* major depressive disorder.

We express our gratitude to Dr. Terao for his detailed commentary^1^ on our article, “Efficacy and Safety of Zuranolone in Japanese Adults with Major Depressive Disorder: A Double‐Blind, Randomized, Placebo‐Controlled, Phase 3 Clinical Trial.”^2^ We have responded to his commentary as follows.

Zuranolone is an orally administered synthetic neuroactive steroid engineered as a modified analog of the endogenous neuroactive steroid allopregnanolone.^3^ Allopregnanolone levels decline following childbirth, a physiological change that is thought to contribute to the onset of postpartum depression (PPD).^4^ In Phase 3 clinical trials for PPD, zuranolone demonstrated statistically significant improvements in depressive symptoms from Day 3 through Day 45, the end of the follow‐up period.^5^, ^6^ Based on these findings, the FDA approved zuranolone as the first orally administered therapy for the treatment of PPD.^7^ While Dr. Terao noted the post‐treatment maintenance of effect in the two phase 3 PPD trials, he conversely suggests there is a loss of effect post‐treatment in major depressive disorder (MDD) based on a lack of significant separation between the zuranolone and placebo groups in change from baseline in the 17‐item Hamilton Rating Scale for Depression (HAM‐D17) total score after the treatment period in this trial.

Although the treatment effect becoming statistically indistinguishable from placebo may be interpreted as a potential loss of relative efficacy, this trial was designed to have sufficient power to detect differences *versus* placebo at Day 15 and might not have been powered to evaluate post‐treatment efficacy; if any; therefore, it has limitations in drawing definitive conclusions regarding post‐treatment effect beyond Day 15, and, importantly, the absence of significant separation from placebo was not the result of symptom worsening in the zuranolone group. The mean HAM‐D17 total scores in both the zuranolone and placebo groups during the follow‐up period (Days 22–57) were numerically lower or comparable to those observed on Day 15. Moreover, the proportion of responders and remitters increased in both groups during the follow‐up period and remained numerically higher for zuranolone at all time points in this trial.^2^, ^8^ Therefore, depressive symptoms improved by the end of the 14‐day treatment with zuranolone and, on average, did not worsen after the treatment period through Day 57.

The differences in whether a statistically significant separation from placebo was achieved during the follow‐up period in the PPD and MDD trials may be attributable to the differences in the magnitude of the antidepressant effects observed during the treatment period. In the Phase 3 clinical trial of PPD, a statistically significant reduction in the HAM‐D17 total score was observed compared with placebo at Day 15, yielding a Cohen's d of 0.52, and this statistical significance was maintained during the follow‐up period.^6^ By contrast, in the present study, a significant reduction in the HAM‐D17 total score at Day15 compared to placebo was observed, but the observed effect size was smaller than in the PPD trial, corresponding to a Cohen's d of 0.21.^2^ The PPD and MDD trials enrolled different patient populations with distinct biological and psychosocial contributors to depression; because PPD represents a more homogeneous subgroup of MDD, comprising women who share a common trigger of pregnancy for their depressive symptoms, this may have contributed to the larger observed effect of zuranolone in PPD. Differences in the magnitude of the treatment effect observed during the treatment period may explain the loss of statistically significant separation from placebo during the follow‐up period in the MDD trial, despite the fact that symptoms did not worsen after treatment discontinuation, similar to what was observed in PPD trials.

In future studies, it will be important to evaluate the long‐term effects of zuranolone, including the identification of background factors associated with its efficacy, as well as whether repeated treatment with zuranolone after the end of the follow‐up period leads to further improvement in symptoms that were maintained after the initial treatment period.

## Disclosure statement

Disclosure statement is listed in Supporting Information.

## Supporting information


**Data S1** Supporting Information.

## Data Availability

Shionogi & Co., Ltd. is committed to disclosing the synopses and results of its clinical trials and sharing clinical trial data (raw dataset or study data tabulation model dataset) with researchers upon reasonable request. If the research proposal is reviewed by an independent review panel and approved, the anonymized data and redacted documents will be provided in a secure research environment. For further details, please refer to the websites of Shionogi & Co., Ltd. (https://www.shionogi.com/global/en/company/policies/shionogi-group-clinical-trial-data-transparency-policy.html) and Vivli (https://vivli.org/).
