# Supplementary material for: Response to Durability of zuranolone in postpartum depression versus major depressive disorder
Source: Psychiatry Clin Neurosci. 2026 May 20;80(7):631–2. doi: 10.1111/pcn.70076 (PMC13332542; doi:10.1111/pcn.70076)
Supplement: Supplementary file 1 — Data S1 Supporting Information. [file PCN-80-631-s001.docx]

**Supplementary: Disclosure statement**

T.S., T.B., and R.S. are full-time employees and own stocks *via* employee stock ownership society of Shionogi & Co., Ltd. T.M. and H.F. are full-time employees of Shionogi & Co., Ltd. J.C.G. is a full-time employee of Shionogi B.V. T.I. has received grants from Daiichi Sankyo, Tsumura, Shionogi & Co., Ltd., Otsuka Pharmaceutical Co., Ltd., Sumitomo Pharma Co., Ltd., Mitsubishi Tanabe Pharma; honoraria for lectures from Mochida Pharmaceutical, Takeda Pharmaceutical Co., Ltd., Janssen Pharmaceuticals, Novartis Pharma, MSD, Yoshitomiyakuhin, Nipro, Kyowa Pharmaceutical Industry, Viatris, Lundbeck Japan K.K., Boehringer Ingelheim, Ono Pharmaceutical, and Meiji Seika Pharma Co., Ltd. , Otsuka Pharmaceutical Co., Ltd., Shionogi & Co., Ltd., Sumitomo Pharma Co., Ltd., Mitsubishi Tanabe Pharma, and Eisai; and is a member of the advisory boards of Luye, Shionogi & Co., Ltd., GlaxoSmithKline, Viatris, and Otsuka Pharmaceutical. M.K. has received grants from AMED and Japanese Ministry of Health, Labour and Welfare; consulting fees from Shionogi & Co., Ltd., Sumitomo Pharma Co., Ltd., Otsuka Pharmaceutical Co., Ltd., Lundbeck Japan K.K., and Takeda Pharmaceutical Co., Ltd.; speaker honoraria from Sumitomo Pharma Co., Ltd., Otsuka Pharmaceutical Co., Ltd., Lundbeck Japan K.K., Takeda Pharmaceutical Co., Ltd., Meiji Seika Pharma Co., Ltd., Shionogi & Co., Ltd., Mitsubishi Tanabe Pharma Corporation, Viatris Inc., Eisai Co., Ltd., and Kyowa Pharmaceutical Industry Co. Ltd.; and is in the general management committee for Depression Treatment Guidelines, Japan Society of Mood Disorder and the Vice Chairman of the Guideline Development Committee Japan Society of Mood Disorders in the past 36 months. K.N. has received grants paid to his institution from Shionogi & Co., Ltd., Sumitomo Pharma Co., Ltd., Otsuka Pharmaceutical Co., Ltd., Janssen Pharmaceutical K.K., Nippon Boehringer Ingelheim Co., Ltd., and AbbVie GK; honoraria from Sumitomo Pharma Co., Ltd. (Speaker, Chair, Advisor), Otsuka Pharmaceutical Co., Ltd. (Speaker, Chair, Advisor), Meiji Seika Pharma Co., Ltd. (Speaker), Janssen Pharmaceutical K.K. (Chair, Speaker-Panelist), Mitsubishi Tanabe Pharma Corp. (Chair, Advisor), Viatris Pharmaceuticals Japan G.K. (Chair, Consulting), Nippon Boehringer Ingelheim Co., Ltd. (Chair, Advisor, Supervisor), Boehringer Ingelheim International GmbH (Speaker-Panelist), Kyowa Kirin Co., Ltd. (Speaker), Shionogi & Co., Ltd. (Chair), and Yoshitomiyakuhin Corp. (Chair, Supervisor); and support for transportation to attend meetings from Sumitomo Pharma Co., Ltd., Otsuka Pharmaceutical Co., Ltd., Meiji Seika Pharma Co., Ltd., Janssen Pharmaceutical K.K., Mitsubishi Tanabe Pharma Corp., Nippon Boehringer Ingelheim Co., Ltd., Boehringer Ingelheim International GmbH, Shionogi & Co., Ltd., Yoshitomiyakuhin Corp., and AbbVie GK.
